# Carbon-ion irradiation overcomes HPV-integration/E2 gene-disruption induced radioresistance of cervical keratinocytes

**DOI:** 10.1093/jrr/rrz048

**Published:** 2019-07-19

**Authors:** Nathalie Arians, Nils Henrik Nicolay, Stephan Brons, Stefan Alexander Koerber, Christine Jaschke, Marco Vercruysse, Sigrid Daffinger, Alexander Rühle, Jürgen Debus, Katja Lindel

**Affiliations:** 1 Department of Radiation Oncology, Heidelberg University Hospital, Im Neuenheimer Feld 400, Heidelberg, Germany; 2 Heidelberg Institute of Radiation Oncology (HIRO), Heidelberg, Germany; 3 National Center for Tumor diseases (NCT), Im Neuenheimer Feld 460, Heidelberg, Germany; 4 Clinical Cooperation Unit Radiation Oncology, German Cancer Research Center (DKFZ), Im Neuenheimer Feld 280, Heidelberg, Germany; 5 Department of Radiation Oncology, Freiburg University Medical Center, Robert-Koch-Straße 3, Freiburg im Breisgau, Germany; 6 Heidelberg Ion-Beam Therapy Center (HIT), Department of Radiation Oncology, Heidelberg University Hospital, Im Neuenheimer Feld 400, Heidelberg, Germany; 7 German Cancer Consortium (DKTK), partner site Heidelberg, Germany; 8 Department of Radiation Oncology, Municipal Hospital Karlsruhe gGmbH, Moltkestraße 90, Karlsruhe, Germany

**Keywords:** human papillomavirus, E2 gene, cervical cancer, radiobiology, radioresistance, heavy-ion therapy

## Abstract

To date, only few data exist on mechanisms underlying the human papillomavirus (HPV)-associated irradiation response. It has been suggested, that the viral E2 gene plays an important role in that context. The aim of the current study is to compare the effect of photon- and carbon-ion (^12^C)-radiation therapy (RT) on cells with different HPV and E2 gene status. We hypothesized that ^12^C-RT might overcome the radioresistance of E2 gene-disrupted cells.

We analyzed four different cell lines that differed in HPV status or E2 gene status. Cells were irradiated with either photons or ^12^C. Clonogenic survival, cell cycle and expression of Rb and p53 were analyzed.

Radiosensitivity seemed to be dependent on E2 gene status and type of RT. ^12^C-RT led to lower surviving fractions, indicating higher radiosensitivity even in cells with disrupted E2 gene. The observed relative biological effectiveness (RBE) of ^12^C-RT for C33a/Caski and W12/S12 was 1.3/4 and 2.7/2.5, respectively. Cell cycle regulation after both photon- and ^12^C-RT was dependent on HPV status and on E2 gene status. Furthermore, the effect of RT on expression of p53 and Rb seemed to be dependent on E2 gene status and type of RT.

We showed that ^12^C-RT overcomes HPV-integration induced radioresistance. The effect of RT on cell cycle regulation as well as on expression of p53 and Rb seemed to be dependent on HPV status, E2 gene status and type of RT. Differences in Rb expression and cell cycle regulation may play a role for enhanced radiosensitivity to ^12^C-RT of cells with disrupted E2 gene.

## INTRODUCTION

Human papillomavirus (HPV)-induced tumors constitute a specific subclass of cancer with a better response to radiation treatment. Previous studies showed that HPV status can be used as a positive predictive marker for treatment outcome for cervical and head and neck cancer patients [1, 2]. However, there are still subgroups of patients showing bad local control and disease-free survival rates after conventional chemoradiotherapy. One reason for increased radioresistance might be bulky disease associated with tumor hypoxia [3]. Furthermore, previous clinical studies demonstrated that the viral E2 gene might play an important role in outcome and local control in cervical carcinomas [4–6]. Additionally, biological experiments have shown that the E2 gene status of HPV 16 influences the radiosensitivity of cervical keratinocytes. Cells with integrated and thus disrupted E2 gene showed a higher radioresistance to irradiation with photons. Cell cycle regulation as well as protein expression of Rb differed in cells with intact or disrupted E2 after radiation treatment [7].

In the course of carcinogenesis, the viral genome gets integrated into the host genome, which frequently leads to disruption of the E2 gene region. Normally, the E2 gene regulates expression of the viral oncogenes E6 and E7, manipulating the cell cycle and apoptosis [8, 9]. The E6 oncoprotein forms a complex with p53, leading to its degradation, and thus overcoming G1/S checkpoint control causing cell cycle dysregulation [10]. The E7 oncoprotein binds to hypophosphorylated Rb resulting in its degradation and inappropriate release of E2F transcription factor [11]. The viral E2 gene regulates viral transcription and genome replication and thus expression of the viral oncogenes E6 and E7, depending on cell type and protein levels [12–15]. Overexpression of E2 has been reported to induce apoptosis [16]. It has also been shown to induce growth arrest in G1-phase of the cell cycle and to abrogate the mitotic checkpoint [17–19]. E2 disruption is not only a result of viral genome integration but can also be caused by high radiation doses [20]. The E2 gene status differs in different cancer stages. In general, integration of E2 is increased in higher cancer stages. Of cervical cancer patients, 17–20% show mixed integrated and episomal forms, 7% show episomal forms only [20].

To date, no satisfying data exist explaining the underlying mechanisms of HPV-associated radiation response. Clinical data regarding primary radiotherapy with ^12^C in advanced cervical cancer patients have already shown favorable local control rates [3, 21, 22]. In this study, we investigated if other radiation modalities like carbon-ions (^12^C) might overcome radioresistance of HPV-integrated and thus E2-disrupted cells. To avoid artificial uncertainties we used the W12/S12 cell model derived from a low-grade cervical lesion by Stanley *et al.* [23] to evaluate the influence of E2 on the intrinsic radiosensitivity of cervical cells to support the hypothesis that ^12^C-RT might overcome radioresistance of E2 gene-disrupted cells. Furthermore, we analyzed cell cycle and protein expression of p53 and Rb to identify molecular mechanisms leading to E2 gene status-dependent differences in radioresistance.

## MATERIALS AND METHODS

### Cell lines and cell culture

Caski cells are an epidermoid cell line derived from a small bowel metastasis of a human cervix carcinoma. The cells contain an integrated HPV-16 genome (about 600 copies per cell) as well as sequences related to HPV-18. C33a cells are an HPV-negative epidermoid cell line which derived from a biopsy of a cervical carcinoma. Rb is present but abnormal in size. p53 expression is elevated and there is a point mutation at codon 273 resulting in an Arg→Cys substitution. The W12 cell line was derived from a low-grade cervical lesion by Stanley *et al.*, and is unique among HPV-16-containing cell lines in carrying its HPV-16 genome as a multicopy episome [23]. S12 cells, which derived from the W12 line, contain HPV DNA as integrated copies [24].

Cells were cultured in Dulbecco’s modified Eagle’s medium (Gibco®, Thermo Fisher Scientific, MA, USA), containing 10% fetal calf serum, 0.5 units penicillin/ml, 0.5 ng streptomycin/ml, 10 ng of cholera toxin/ml, 0.5 μg of hydrocortisone/ml and 10 ng of epithelial growth factor per ml (Sigma, St. Louis, MO, USA). Cells were split at 80% confluence. W12 cells were cultured with lethally irradiated Swiss 3T3 feeder cells. S12 cells were obtained by collecting surviving W12 cells cultured without feeder-layer support.

The HPV16-positive cells were tested for an intact E2 gene in three separate amplification reactions which allows the amplification of three amplicons of different length, determining the integrity of the E2 gene [4]. Details of the procedure have been described previously [7].

### Clonogenic growth assay and irradiation

Clonogenic survival was performed in 96-well plates: 1–100 cells per well were seeded. The plates were examined with an inverted phase-contrast microscope at intervals of 7, 10 and 14 days. A well was considered positive when a colony in it reached a size of ≥50 cells. The cells were fixed with 70% ethanol for 10 min prior to staining with 0.1% methylene blue. After staining wells were washed with distilled water. Plating efficiency (PE) was calculated using Poisson statistics according to the formula PE = −ln (negative wells/total wells)/number of cells plated per well [25].

Cells were irradiated with single doses of 0, 2 and 7 Gy photons or 0, 0.5, 1 and 2 Gy ^12^C. In such experiments, an increasing number of cells was plated for each increment in irradiation dose. Therefore, the effect of cell number per well on plating efficiency was evaluated. Survival curves were based on the number of positive wells or colonies in each irradiated group as a fraction of that in the unirradiated group.

Photon irradiation was performed with a biological cabinet X-ray irradiator (XRAD 320 Precision X-ray Inc., N. Bradford, CT) at single doses of 0, 2 and 7 Gy. Specifications of the cabinet X-ray irradiator according to the manufacturer are the following. Maximum potential of the x-ray tube: 320 kV. Dose output: 3 Gy/min at 320 kV, 12.5mA, 50 cm Source-to-surface distance (SSD) (half-value layer [HVL] ≈ 1 mm Cu); 1 Gy/min at 320 kV, 12.5 mA, 50 cm SSD, (HVL ≈ 4 mm Cu). Radiotherapy was performed at room temperature.

Carbon-ion radiotherapy was performed at the Heidelberg Ion-Beam Therapy Center with the horizontal beamline using the raster scanning technique developed by Haberer *et al.* [26]. Single doses of 0, 0.5, 1 and 2 Gy were delivered with an extended Bragg peak (dose average linear energy transfer (LET), 103 keV/μm) that was adjusted using a 3 cm acrylic shield and positioning cell monolayers in the middle of the extended Bragg peak.

RBE (relative biological effectiveness) of ^12^C-RT was calculated by comparing the radiation dose at 10% cell survival with the radiation dose of photon-RT at 10% cell survival for all cell lines. The RBE takes into account different biological effects of the same physically absorbed dose for different radiation modalities.

Furthermore, the REF (radiation enhancement factor) was calculated by comparing the radiation dose at 10% cell survival of W12 (E2 intact) vs S12 (E2 disrupted) cells. The REF is similar to other radiobiological ratios (such as oxygen effect, RBE). In our study, the REF represents the influence of E2-gene status on radiosensitivity. The REF shows the ratio of the dose leading to 10% cell survival in W12 divided by the dose leading to 10% cell survival in S12 cells.

$$\mathrm{REF}=\frac{\mathrm{dose}\;\;\mathrm{at}\;\mathrm{10}\%\;\;\mathrm{cell}\;\mathrm{survival}\;\mathrm{in}\;\mathrm{W12}\;\left(\mathrm{E2}\;\mathrm{gene}\;\mathrm{intact}\right)}{\mathrm{dose}\;\;\mathrm{at}\;\;\mathrm{10}\%\;\mathrm{cell}\;\mathrm{survival}\;\mathrm{in}\;\;\mathrm{S12}\;(\mathrm{E2}\;\mathrm{gene}\;\mathrm{disrupted})}$$

### Cell cycle analyses

Cell cycle analyses were performed 0, 24, 48, 72 h after irradiation with 2 and 7 Gy photons and 0.5, 1 and 2 Gy ^12^C by flow cytometry using propidium iodide (PI)-staining as described elsewhere [27]. Data were collected by using FACScan flow cytometry, and results were analyzed by using Cellquest software (Becton Dickinson, Franklin Lakes, NJ, USA). For each sample, 10 000 events were collected, and aggregated cells were gated out.

### Intracellular cytokine staining of Rb and p53

The retinoblastoma gene encodes a nuclear phosphoprotein that is expressed in most normal cells and acts as a tumor suppressor. An under-phosphorylated form of Rb binds to the viral oncogene HPV-E7 [8]. Clone G3-245 recognizes an epitope between amino acids 300 and 380 of the human retinoblastoma protein (pp110-114 Rb).

Wildtype p53 forms specific complexes with several viral oncogenes including HPV-E6 and plays a role as a checkpoint protein for DNA damage during the G1/S-phase of the cell cycle. Clone G59-12 recognizes mutant and wild type human, mouse and rat p53 suppressor protein.

The G3-245 or G59-12 and MOPC-21 FITC (a mouse IgG1 isotype control) conjugates are matched and fluorochrome/protein ratios determined experimentally by flow cytometric analysis.

### Details of the procedure

Cells were fixed in 70% ethanol and washed twice in cold PBS, then resuspended in fixation/permiabilisation solution Perm/Wash^TM^ (BD Biosciences, Heidelberg, Germany) (1×10^6^ cells/ml) for 30 min at 4°C and pelleted by centrifugation. Afterwards buffer was removed and cells were washed twice in fresh Perm/Wash^TM^ BD buffer. Thoroughly resuspended cells were subjected to intracellular cytokine staining by incubating in 100 μl of Perm/Wash^TM^ BD buffer containing 20 μl of Fluorochrome-conjugated Rb-antibody (FITC mouse antihuman retinoblastoma antibody from Becton Dickinson, Franklin Lakes, NJ, USA) for 24 h at 4°C temperature in the dark. After washing with Perm/Wash^TM^ BD cells were pelleted and resuspended in 0.5 ml of Perm/Wash^TM^ BD for flow cytometric analysis. The same procedure was performed for p53 staining using 20 μl of Fluorochrome-conjugated antibody p53-ak (FITC mouse anti-human p53 antibody from Becton Dickinson, Franklin Lakes, NJ, USA)

### Flow cytometric analysis

Stained cells were analyzed using FACSCan flow cytometry (BD Biosciences, Heidelberg, Germany) equipped with an air-cooled 488 nm argon-ion laser. Data acquisition and analysis were performed using FACSComp and Cellquest (version 3.4) software. A total event of 10 000 cells were acquired for each sample. Data were expressed as geometric mean fluorescence intensity and as the ratio between the fluorescence emission of sample cells and that of the isotypic control (P/N ratio; positive/negative). In each case negative controls were cells treated as described above without Rb-ak staining or p53-ak staining. Isotypic controls were cells treated with an isotype-matched control of irrelevant specificity from FITC Mouse IgG1 Isotype control (Becton Dickinson, Franklin Lakes, New Jersey, USA) instead of Rb-ak staining or p53-ak staining. Analyses were performed after 0 and 24 h of irradiation.

### Statistical analysis

For data quantification, mean values and standard deviation were calculated from at least three experimental replicates. Data are shown as mean values ± standard deviation. Survival curves were generated with the linear quadratic (LQ) model. Sigma plot’s (Systat Software GmbH, Erkrath, Germany) non-linear least-squares regression option was used to fit the calculated survival curves. RBE and REF were calculated by comparing the radiation dose at 10% cell survival. Statistical analyses were performed using the one sample *t*-test, and two-sided *P*-values < 0.05 were considered significant.

## RESULTS

**Fig. 1.**
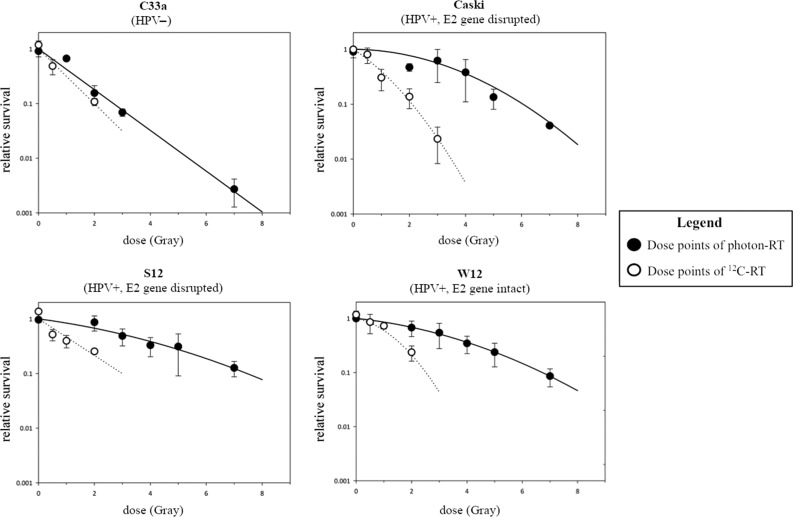
Clonogenic survival after photon- and ^12^C-RT. REF for photon-RT W12 vs S12 cells was 1.1 and Caski vs C33a was 2.3. REF for ^12^C-RT W12 vs S12 was 0.9 and Caski vs C33a was 0.7.

W12 (intact E2 gene) showed lower surviving fractions than S12 (disrupted E2 gene) after photon-RT. The photon radiation dose leading to 10% cell survival was 1.1-fold higher in S12 cells compared with W12 cells, indicating that an intact E2 gene enhances radiosensitivity to photons 1.1-fold (REF of 1.1). After ^12^C-RT, W12 as well as S12 showed a strong decrease in surviving cells with a RBE of ^12^C-RT of 2.7 and 2.5, respectively. Caski (HPV+, E2 gene disrupted) showed higher surviving fractions after photon-RT than C33a (HPV-). After ^12^C-RT, Caski and C33a showed significantly lower surviving fractions compared with photon-RT with an RBE of ^12^C-RT of 4.3 and 1.3, respectively (Fig. 1).

**Fig. 2.**
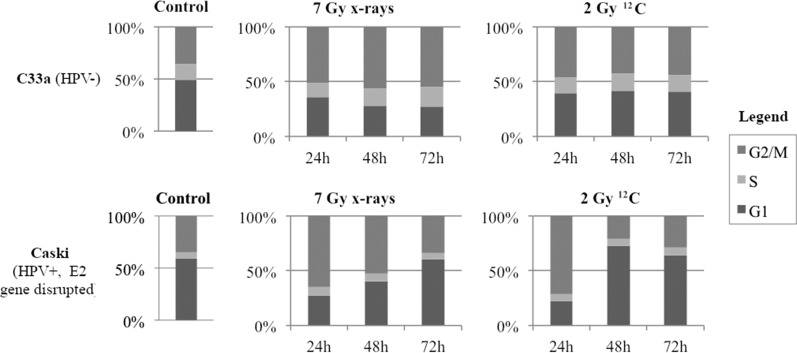
Cell cycle analyses of HPV-negative (C33a) and HPV-positive (Caski) cells after irradiation with 7 Gy photons and 2 Gy ^12^C after 24, 48 and 72 h, respectively.

**Fig. 3.**
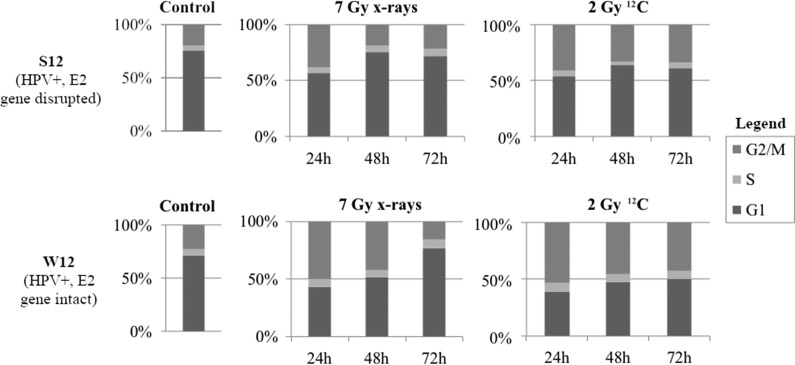
Cell cycle analyses of HPV-integrated (S12) and HPV-episomal (W12) cells after irradiation with 7 Gy photons and 2 Gy ^12^C after 24, 48 and 72 h, respectively.

In C33a, G2/M-block was only induced after irradiation with higher radiation doses: 24 h after irradiation with 7 Gy photons/2 Gy ^12^C, the amount of cells in the G2/M-phase increased to 49.9%/44.4 %, respectively, compared with 34.9% in the control group. Caski showed a G2/M-block after irradiation with both photons and ^12^C. At 24 h after irradiation with 2/7 Gy photons the amount of cells in G2/M-phase increased to 51%/56% compared with 35.1% in the control group. After irradiation with 0.5/2 Gy ^12^C, the amount of cells in G2/M-phase after 24 h increased to 60.2%/71.4%, respectively. (Fig. 2 and see online Supplementary material). W12 with intact E2 gene showed a G2/M-block after irradiation with both photons and ^12^C. At 24 h after irradiation with 2/7 Gy photons the amount of cells in G2/M-phase increased to 29.8%/44.6%, compared with 21% in the control group. After irradiation with 0.5/1/2 Gy ^12^C the amount of cells in G2/M-phase after 24 h increased to 28.9%/25.7%/46.4%, respectively. S12 showed a G2/M-block after irradiation with higher photon doses and after ^12^C-RT. At 24 h after irradiation with 2/7 Gy photons the amount of cells in G2/M-phase increased to 21.8%/35.5%, compared with 18.9 % in the control group. After irradiation with 0.5/1/2 Gy ^12^C, the amount of cells in G2/M-phase after 24 h increased to 26.5%/31.9%/39%, respectively (Fig. 3 and online Supplementary material).

**Fig. 4.**
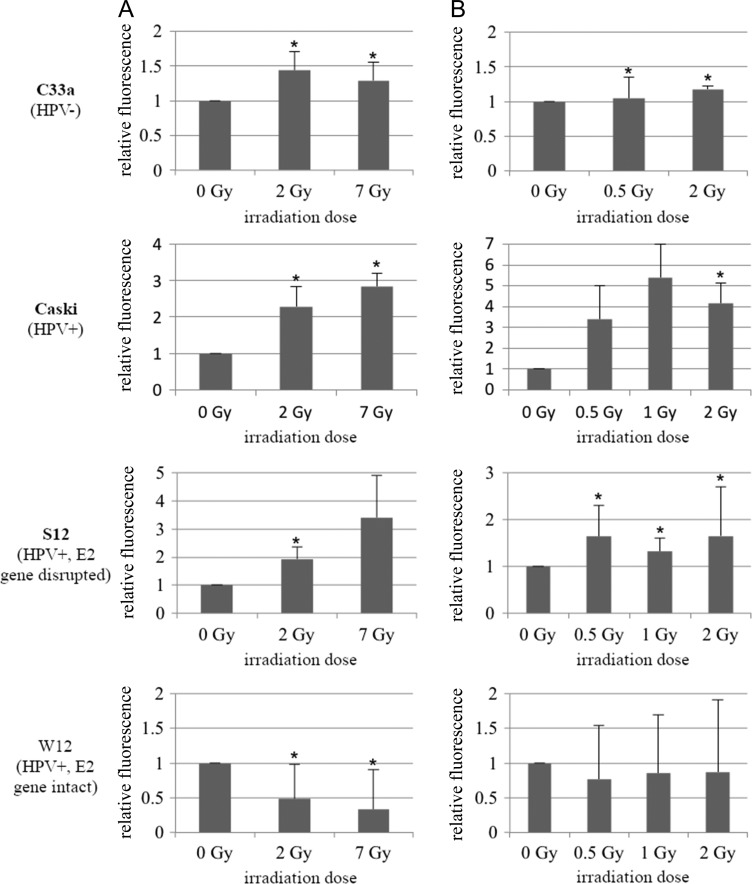
Expression of p53 after [**A**] photon-RT and [**B**] ^12^C-RT. Expression of p53 was analysed 24 h after irradiation with either photons or ^12^C. C33a: 2/7 Gy photons → 1.4-fold (*P* = 0.0035)/1.3-fold (*P* = 0.0037) increase. 0.5/2 Gy ^12^C → 1.1-fold (*P* = 0.0066)/1.2-fold (*P* = 0.0001) increase. Caski: 2/7 Gy photons → 2.3-fold (*P* = 0.0097)/2.8-fold (*P* = 0.003) increase. 0.5/1/2 Gy ^12^C → 3.4-fold (*P* = 0.0508)/5.4-fold (ns)/4.2-fold (*P* = 0.0259) increase. W12: 2/7 Gy photons → 0.5-/0.3-fold decrease (ns). 0.5/1/2 Gy ^12^C → 0.8-/0.9-/0.9-fold decrease (ns). S12: 2/7 Gy photons → 1.9-fold (*P* = 0.00722)/3.4-fold (*P* = 0.0526) increase. 0.5/1/2 Gy ^12^C → 1.6-fold (*P* = 0.0178)/1.3-fold (*P* = 0.0043)/1.7-fold (*P* = 0.0292) increase. ns = not significant.

**Fig. 5.**
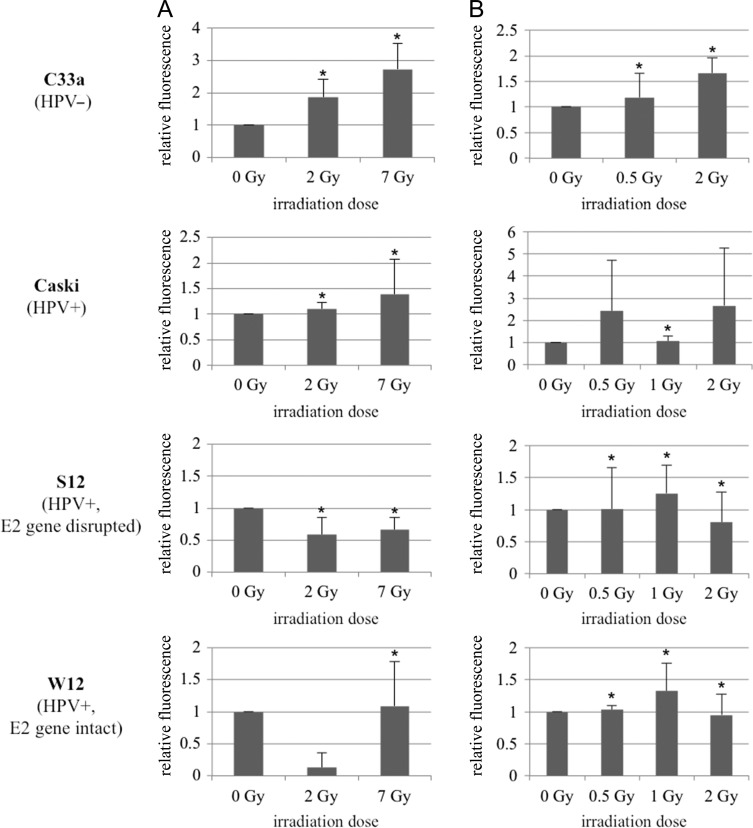
Expression of Rb after [**A**] photon-RT and [**B**] ^12^C-RT. Expression of Rb was analysed 24 h after irradiation with either photons or ^12^C. C33a: 2/7 Gy photons → 1.9-fold (*P* = 0.0^12^3)/2.7-fold (*P* = 0.0199) increase. 0.5/2 Gy ^12^C → 1.2-fold (*P* = 0.0097)/1.7-fold (*P* = 0.0034) increase. Caski: 2/7 Gy photons → 1.1-fold (*P* = 0.0008)/1.4-fold (*P* = 0.0171) increase. 0.5/1/2 Gy ^12^C → 2.4-fold (ns)/1.1-fold (*P* = 0.0026)/2.7-fold (ns) increase. W12: 2/7 Gy photons → 0.13-fold/1.1-fold (*P* = 0.0214) increase. 0.5/1/2 Gy ^12^C → 1.01-fold (*P* = 0.0003)/1.3-fold (*P* = 0.0073)/1.0-fold (*P* = 0.006) increase. S12: 2/7 Gy photons → 0.6-fold (*P* = 0.0108)/0.7-fold (*P* = 0.0048) decrease. 0.5/1 Gy ^12^C → 1.01-fold (*P* = 0.0193)/1.3-fold (*P* = 0.0119) increase. 2 Gy ^12^C → 0.8-fold decrease (*P* = 0.0149). ns = not significant.

Figures 4 and 5 show expression of p53 and Rb 24 h after photon- and ^12^C-RT. After both photon- and ^12^C-RT, C33a showed slightly but significantly increased p53-expression. (1.4-/1.3-fold after 2/7 Gy photons; 1.1-/1.2-fold after 0.5/2 Gy ^12^C). Caski showed a stronger and significant increase in p53 expression after both photon- and ^12^C-RT (2.3-/2.8-fold after 2/7 Gy photons; 4.2-fold after 2 Gy ^12^C). After both photon- and ^12^C-RT, C33a showed significantly increased Rb expression (1.9-/2.7-fold after 2/7 Gy photons; 1.2-/1.7-fold after 0.5/2 Gy ^12^C), which was even stronger compared with Caski (1.1-/1.4-fold after 2/7 Gy photons and 1.1-fold after 1 Gy ^12^C).

In general, W12 showed a very low expression of p53. After irradiation with 2/7 Gy photons, p53-expression significantly decreased (0.5-/0.3-fold). After irradiation with 0.5/1/2 Gy ^12^C W12 only showed a trend for a decrease in p53-expression (0.8-/0.9-/0.9-fold), compared with the unirradiated control. In contrast, S12 showed a statistically significant upregulation of p53 after both photon- and ^12^C-RT (1.9-fold after 2 Gy photons; 1.6-/1.3-/1.7-fold after 0.5/1/2 Gy ^12^C). W12 had also lower baseline-expression of Rb than S12. Rb slightly but significantly increased 24 h after 7 Gy photon- (1.1-fold) and after ^12^C-RT (1.01-/1.3-fold increase after 0.5/1 Gy). In contrast, S12 showed a statistically significant decrease in Rb expression after irradiation with photons (0.6-fold/0.7-fold after 2/7 Gy photons). Interestingly, after irradiation with 0.5/1Gy ^12^C, S12, like W12, showed 1.01-/1.3-fold increased Rb expression. After irradiation with 2 Gy ^12^C S12 showed slightly but significantly decreased Rb-levels (0.8-fold).

## DISCUSSION

Our results for the clonogenic survival analyses showed that HPV-positive cancer cells with disrupted E2 gene are more radioresistant than HPV-negative cancer cells. This effect seemed to be abolished by the use of ^12^C-RT. We established the W12/S12 cell system, mimicking the natural way of integration of virus DNA leading to disruption of the E2 gene [7, 23, 24], to further analyse the influence of the E2 gene status on radiosensitivity. ^12^C-RT induced a strong decrease in surviving fraction in cells with disrupted E2 gene, in contrast to photon-RT. Thus, irradiation with ^12^C bears the potential to overcome HPV-integration-induced radioresistance.

In general, cells with disrupted E2 gene showed a much higher proportion of cells in the G1-phase, which might be one explanation for the generally lower radiosensitivity. However, all cell lines failed to arrest in the G1-phase after irradiation of both kinds, independent of E2 gene status. DeWeese *et al.* previously showed that irradiation leads to only G2 arrest and no G1 arrest in cells expressing the viral oncoproteins E6 and E7 [28], which is consistent with our data. In our HPV-positive cells, irradiation of both kinds induced a G2/M-block, which was more pronounced after ^12^C-RT. Ziemann *et al.* showed an increased G2/M-block of HPV-positive tumor cells after photon-RT compared with HPV-negative cells, which was associated with a better response to radiotherapy in that study [29]. This difference might be explained by the use of different HPV-positive cells. We showed that the observed G2/M-block in HPV-positive cancer cells was dependent on E2 gene status. In cells with intact E2 gene, G2/M-block appeared earlier, stronger and lasted longer (up to 48 h after photon- and 72 h after ^12^C-RT) compared with cells with disrupted E2 gene (up to 24 h after photon- and 72 h after ^12^C-RT) (see Fig. 3). Previous data showed that radiosensitive cells develop a longer G2/M-block after a specific radiation dose that matches normal or resistant cells [30], which confirms our observations. However, we showed that ^12^C-RT induced a G2/M-block in E2-disrupted cells, which was not as strong as the G2/M-block in E2-intact cells but also lasted for 72 h, possibly leading to the enhanced radiosensitivity of E2-disrupted cells to ^12^C-RT compared with photon-RT.

Normally, DNA damage leads to increased p53 levels resulting in G1/S-arrest. Loss of expression of p53 or expression of mutant p53 results in failure of G1-arrest [31, 32]. C33a cells show a point mutation in the p53 gene, leading to expression of dysfunctional p53. Rb is present, but abnormal in size. These observations may explain the lack of G1- or G2/M-arrest after irradiation. In HPV-positive cells the E6 oncoprotein forms a complex with p53, leading to its degradation, thus overcoming cell cycle checkpoint control and causing cell cycle dysregulation [10]. It has been reported, that irradiation enhances the expression of the viral oncoproteins E6 and E7, which controversially doesn’t result in lower p53-levels [29]. In our HPV-positive cells, irradiation led to increased p53-levels after photon- and ^12^C-RT, depending on E2 gene status. ^12^C-RT induced higher p53-levels, resulting in a more pronounced G2/M-block.

Furthermore, in HPV-positive cells the E7 oncoprotein acts as inhibitor of Rb, also resulting in dysfunctional cell cycle control [11]. Rb expression seemed to be dependent on both E2 gene status as well as on type of RT. Baseline Rb-expression was higher in cells with disrupted E2 gene. Photon-RT led to a significant decrease in Rb levels in cells with disrupted E2 gene. There are data showing increased expression of E6/E7 in cervical cancer cells after photon-RT [33], which normally results in lower Rb-levels. Furthermore, expression of E7 is dependent on E2 gene status. It has been stated previously, that the disrupted E2 gene in W12 cells leads to overexpression of E7 and thus degradation of Rb after photon-RT [7]. In our study, ^12^C-RT on the other hand led to increased Rb-levels, even in cells with integrated E2 gene. This might explain the more pronounced G2/M-block after ^12^C-RT and thus higher radiosensitivity to ^12^C-RT compared with photon-RT.

In summary, our experiments confirm an irradiation-induced G2/M-arrest in HPV-positive cells, depending on the E2 gene status. After ^12^C-RT also, cells with disrupted E2 gene showed a G2/M-block, which might be one possible mechanism for the enhanced radiosensitivity to ^12^C-RT compared with photon-RT. Consistent with this hypothesis is the E2-dependent p53-expression and RT-dependent Rb-expression in E2 gene-disrupted cells. Additionally, our experiments confirm a lack of G1-arrest independent of E2 status.

However, there might be other mechanisms underlying the higher radiosensitivity to ^12^C-RT of cells with disrupted E2 gene. It has been reported previously, that HPV-positive cells have an impaired double-strand break repair and tend to accumulate double-strand breaks during the cell cycle [34, 35]. Further experiments are necessary to investigate the role of disrupted E2 gene on DNA repair mechanisms. We hypothesize that cells with disrupted E2 gene have a higher capability of repairing sublethal DNA-damage. Generally, irradiation with heavy ions generates more lethal DNA-damage, which the cell is unable to repair leading to cell death resulting in the lower surviving fractions after ^12^C-RT even in cells with disrupted E2 gene.

We have shown that ^12^C-RT overcomes HPV-integration induced radioresistance. Differences in Rb expression and cell cycle regulation may play a role in enhanced radiosensitivity to ^12^C-RT of cells with disrupted E2 gene. Understanding the molecular mechanisms responsible for the better treatment response of patients with HPV-positive cervical cancer with intact E2 gene is essential to adapt current treatment strategies and to develop individualized, risk-adapted approaches.

## Supplementary Material

rrz048_Supplementary
