# Supplementary figures and images for: Carbon-ion irradiation overcomes HPV-integration/E2 gene-disruption induced radioresistance of cervical keratinocytes
Source: J Radiat Res. 2019 Jul 19;60(5):564–72. doi: 10.1093/jrr/rrz048 (PMC6805985; doi:10.1093/jrr/rrz048)

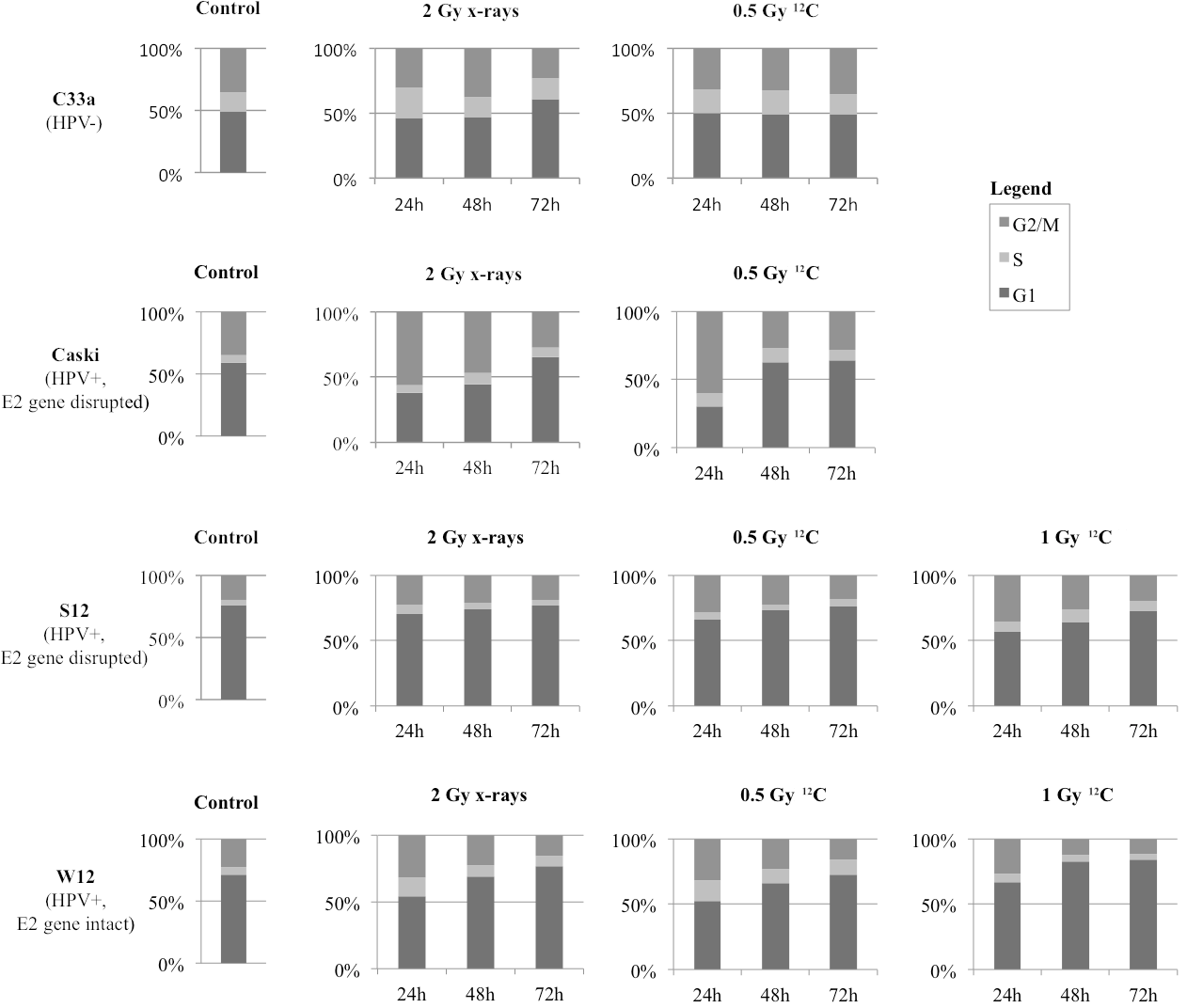

Supplement: rrz048_Supplementary [file rrz048_supplementary.png]
